# Structural and functional insights into the reaction specificity of catalase-related hydroperoxide lyase: A shift from lyase activity to allene oxide synthase by site-directed mutagenesis

**DOI:** 10.1371/journal.pone.0185291

**Published:** 2017-09-27

**Authors:** Tarvi Teder, Helike Lõhelaid, Nigulas Samel

**Affiliations:** Department of Chemistry and Biotechnolgy, Tallinn University of Technology, Tallinn, Estonia; Universidade Nova de Lisboa Instituto de Tecnologia Quimica e Biologica, PORTUGAL

## Abstract

Two highly identical fusion proteins, an allene oxide synthase-lipoxygenase (AOS-LOX) and a hydroperoxide lyase-lipoxygenase (HPL-LOX), were identified in the soft coral *Capnella imbricata*. Both enzymes initially catalyze the formation of 8*R*-hydroperoxy-eicosatetraenoic acid (8*R*-HpETE) from arachidonic acid by the C-terminal lipoxygenase (LOX) domain. Despite the fact that the defined catalytically important residues of N-terminal catalase-related allene oxide synthase (cAOS) domain are also conserved in *C*. *imbricata* hydroperoxide lyase (cHPL), their reaction specificities differ. In the present study, we tested which of the amino acid substitutions around the active site of cHPL are responsible for a control in the reaction specificity. The possible candidates were determined via comparative sequence and structural analysis of the substrate channel and the heme region of coral cAOSs and *C*. *imbricata* cHPL. The amino acid replacements in cHPL—R56G, ME59-60LK, P65A, F150L, YS176-177NL, I357V, and SSSAGE155-160PVKEGD—with the corresponding residues of cAOS were conducted by site-directed mutagenesis. Although all these mutations influenced the catalytic efficiency of cHPL, only F150L and YS176-177NL substitutions caused a shift in the reaction specificity from HPL to AOS. The docking analysis of *P*. *homomalla* cAOS with 8*R*-HpETE substrate revealed that the Leu150 of cAOS interacts with the C5-C6 double bond and the Leu177 with the hydrophobic tail of 8*R*-HpETE. We propose that the corresponding residues in cHPL, Phe150 and Ser177, are involved in a proper coordination of the epoxy allylic radical intermediate necessary for aldehyde formation in the hydroperoxide lyase reaction.

## Introduction

Oxylipins are oxidized polyunsaturated fatty acids and their biosynthesis can be divided into two main steps [1]. Firstly, a molecular oxygen is introduced to a polyunsaturated fatty acid substrate by a dioxygenase resulting in the formation of a primary oxylipin (fatty acid hydroperoxide) [2,3]. Secondly, the rearrangement of oxygens or the modification of a functional group of a primary oxylipin is catalyzed by various enzymes, mostly related to the cytochrome P450 superfamily [4]. In animals, thromboxane synthase (CYP5) or prostacyclin synthase (CYP8) are responsible for the synthesis of the corresponding lipid mediators from cyclooxygenase-derived endoperoxides [5]. In plants, lipoxygenase-derived fatty acid hydroperoxides are mainly converted by different members of the cytochrome P450 CYP74 family [6]. For example, allene oxide synthase (AOS) pathway is involved in the synthesis of an important plant stress hormone, jasmonic acid [7], and hydroperoxide lyase (HPL) gives rise to short-chain aldehydes with antifungal and -bacterial properties [8].

**Fig 1:**
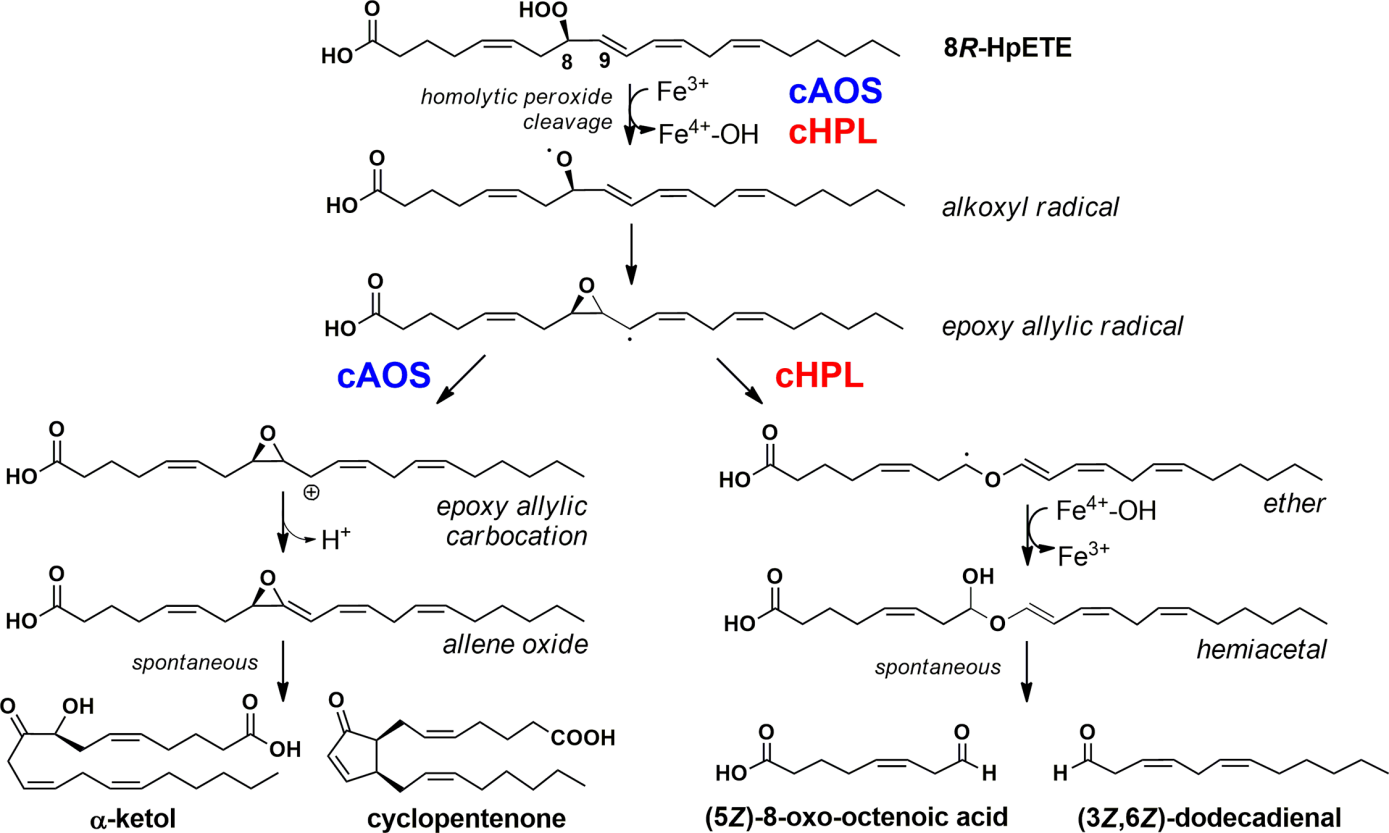
Two parallel 8*R*-HpETE-dependent pathways in the soft coral *C*. *imbricata*. Highly identical cAOS and cHPL domains both catalyze the formation of an epoxy allylic radical intermediate but the next steps in the corresponding reactions differ. The accepted way in presenting cAOS reaction is via a carbocation intermediate while cHPL reaction can be described by using a radical pathway [14].

In soft corals (*Cnidaria*, *Animalia*), a lipoxygenase (LOX) and an allene oxide synthase (AOS) are encoded as a single fusion protein having the ability to catalyze two sequential reactions [9,10]. Recently, in the soft coral *Capnella imbricata*, an AOS-LOX fusion protein was identified in parallel with another fusion protein, a hydroperoxide lyase-lipoxygenase (HPL-LOX) [11]. The C-terminal LOX domain of both fusion proteins initially converts arachidonic acid (AA) to 8*R*-hydroperoxy-eicosatetraenoic acid (8*R*-HpETE) while the reaction specificities of N-terminal catalase-related AOS (cAOS) and HPL (cHPL) domains differ [12]. The cAOS domain synthesizes an unstable allene-8,9-epoxide (AO), detected as its stable hydrolysis products, α-ketol and cyclopentenone, whereas the cHPL domain produces short-chain aldehydes, 8-oxo-(6*E*)-octenoic acid (C8-oxo acid) and (3*Z*,6*Z*)-dodecadienal (C12 aldehyde) (Fig 1) [12]. Previously, we have shown that the gene expression and eicosanoid synthesis of *C*. *imbricata* cAOS-LOX increased in response to abiotic stressors like mechanical injury [11] and elevated water temperature [13] while the biological role of cHPL-LOX remained elusive.

Despite the fact that allene oxides are also formed by plant and fungal cytochrome P450s [15–17], these enzymes are not structurally related to coral cAOS [18]. Instead, the crystal structure of *Plexaura homomalla* cAOS revealed a similar core structure to catalase. Moreover, the heme coordinating residues, R64, R102, R360; the distal heme residues, H67, T66, N137; and the proximal heme ligand, Y353 of cAOS, are identical to the corresponding residues of catalase [18]. Despite these similarities between cAOS and catalase, the former is not able to scavenge hydrogen peroxide (H_2_O_2_) [18].

The structural comparison of highly conserved cAOS and cHPL domains revealed some variations in the substrate channel and the heme region. In the present study, using *in silico* modelling and mutational analysis we attempted to find out which of these substitutions were responsible in the cHPL-specific reaction.

## Materials and methods

### Materials

AA, [1-^14^C]-labeled AA and H_2_O_2_ were purchased from Cayman Chemical Co., GE Healthcare and Merck, respectively. 8*R*-HpETE and [1-^14^C]-labeled 8*R*-HpETE were synthesized using the *C*. *imbricata* 8*R*-lipoxygenase domain of the cHPL-LOX fusion protein, expressed in *Escherichia coli*. Only HPLC grade solvents (Sigma-Aldrich) were used. All the other reagents were purchased from Merck.

### Sequence and structural analysis of coral cAOS and cHPL

Amino acid sequences were aligned using MegAlign software (DNAStar 7.1). 3D models of cHPL and its mutants were prepared based on the *P*. *homomalla* cAOS (PDB code: 1u5u) and cAOS-LOX (PDB code: 3dy5) crystal structures using CPHmodels 3.2 server [19]. The high sequence identity between the template and targets around 83% (S1A Fig) and the sufficient resolution of X-ray structures, 2.0 Å and 3.5 Å for *P*. *homomalla* cAOS and cAOS-LOX, respectively, were preconditions for the homology models with a good quality. As the protein crystals were grown around pH 6, the obtained models also contained positively charged Lys and Arg, and negatively charged Glu and Asp residues [18]. The surface area and the volume of binding pockets of proteins were calculated using CASTp server and are presented as Å^2^ and Å^3^, respectively [20]. The docking of *P*. *homomalla* cAOS with two ligands, 8*R*-HpETE or AO, was conducted using the Swissdock tool [21]. The carboxy groups of fatty acids are negatively charged at a pH greater than 4.5, however, identical docking results were obtained with either charged or neutral ligands. Protein structures were visualized using UCSF Chimera (1.10.1) software [22].

### Preparation of expression constructs

The R56G (Arg56 in cHPL; Gly56 in cAOS), P65A, ME59-60LK, F150L, YS176-177NL, I357V mutations were inserted into the cHPL-His_4_+pET11a construct [12] by using the whole plasmid PCR method [23]. Complementary primers for the site-directed mutagenesis with the silent mutations for the restriction analysis are presented in S1 Table. The PVKEGD fragment was PCR-amplified from the His_6_-cAOS-LOX sequence [11] by specific forward- and reverse primers (S1 Table) with *Xho*I and *Psi*I restriction sites, respectively. For the SSSAGE155-160PVKEGD replacement, the *Xho*I restriction site was introduced to the cHPL-His_4_+pET11a construct by using the whole plasmid PCR method as described above. The SSSAGE fragment in the cHPL sequence was replaced with the PVKEGD fragment from the cAOS sequence. The ORF of the 8*R*-LOX domain with an N-terminal His_6_-tag was PCR-amplified from the His_6_-cHPL-LOX sequence [11] and cloned into the pET11a expression vector (Stratagene). Similarly, the *C*. *imbricata* cAOS-H4 domain was PCR-amplified from the His_6_-cAOS-LOX sequence and cloned into the same expression vector. The cAOS L150F construct was derived from the cAOS-H4 sequence by using the whole plasmid PCR method as described above. The primers for His_6_-8*R*-LOX, cAOS-H4, and cAOS L150F used in PCR are presented in S1 Table. All the mutations were screened by restriction analysis and all the prepared constructs were sequenced (Agowa, Germany).

### Expression and purification of cHPL mutants

Expression vectors were transformed into *E*. *coli* BL21(DE3) cells (Novagen) and the expression at OD_600_ = 0.7–0.8 was induced with 1-thio-β-D-galactopyranoside (IPTG; final concentration at 0.4 mM). Most of the mutants were expressed in 300 mL of Terrific Broth (TB) medium at 15°C overnight. The cAOS L150F and cHPL SSSAGE155-160PVKEGD were expressed in 1.2 and 2.4 L of TB medium, respectively, at 10°C for 3 days. The *C*. *imbricata* 8*R*-LOX and cAOS-H4 domains were expressed in 300 mL and 3 L of TB medium, respectively, at 10°C overnight. All the cultures were harvested and stored at -80°C. The cell pellets were resuspended in a 20 mM Tris (pH 8.0) buffer containing 100 μM phenylmethylsulfonyl fluoride, 0.2% Tergitol NP-40 (Sigma-Aldrich) and 1 mg/mL of lysozyme (Sigma-Aldrich), and sonicated using a Torbeo 36810-series ultrasonic cell disruptor (Cole Parmer). The proteins recovered in the 40 000 x g supernatant of sonicated cells were loaded on the nickel-NTA column (0.5 ml of bed volume, Sigma-Aldrich) equilibrated with the loading buffer (20 mM Tris, 300 mM NaCl, pH 8.0). The His-tagged proteins were eluted with the elution buffer (20 mM Tris, 300 mM NaCl, 200 mM imidazole, pH 8.0) and the 0.5 mL fractions were collected and assayed for activity. The positive fractions were dialyzed against a 50 mM Tris (pH 8.0) and 150 mM NaCl buffer by using an OrDial14 regenerated cellulose tubular dialyzis membrane (MWCO: 12000–14000 Da; Orange Scientific) by slowly stirring at 4°C overnight. The dialyzed proteins were stored at -80°C for further use. The purity of protein preparations was determined by SDS-PAGE. All the mutants were quantified at 406 nm (ε ~ 100 000 M^-1^ cm^-1^) characteristic for hemoproteins. The wt cHPL and mutants with a same heme concentration were compared by SDS-PAGE and the relative quantities of heme-free protein were determined by using GeneTools densitometry (Syngene).

### Incubations with the 8*R*-HpETE substrate

Incubations with each cHPL mutant (10 nM) were performed using 10 μM 8*R*-HpETE in 50 mM Tris (pH 8.0) buffer containing 100 mM NaCl and 1 mM CaCl_2_ in a quartz cuvette with constant stirring at 20°C. The disappearance of a conjugated diene chromophore (ε ~ 25 000 M^-1^ cm^-1^) at 235 nm was recorded using a 1601 UV-visible spectrometer (Shimadzu). The kinetic measurements were conducted using 5–50 μM 8*R*-HpETE substrate and the initial reaction velocity was determined from the linear part of the curve. The k_cat_ and K_m_ values were calculated employing the nonlinear regression analysis of the Michaelis-Menten equation.

Incubations with 20 μM [1-^14^C]-AA-derived 8*R*-HpETE were performed for the product analysis. The reactions were stopped using a mild reducing agent SnCl_2_ (final concentration at 1 mg/mL) and acidified with HCl to pH 3.5. The products were extracted using ethyl acetate, taken to dryness and dissolved in an HPLC eluent prior to further analysis.

### Incubations with the H_2_O_2_ substrate

Incubations with 60 nM wt cHPL or selected mutants were conducted using 0.8 mM H_2_O_2_ substrate in a 50 mM Tris (pH 8.0) buffer containing 100 mM NaCl and 1 mM CaCl_2_ in a cuvette with constant stirring at 20°C. Incubations with the *C*. *imbricata* cAOS-LOX and the cAOS domain were conducted in parallel. The production of oxygen was measured with the Model 110 Fiber Optic Oxygen Monitor (Instech) and the k_cat_ values were determined based on the initial production of oxygen in μM/min.

### RP-HPLC-MS analysis of products formed by cHPL mutants

The products were analyzed by RP-HPLC connected to MSMS or radiometric detector by using the same protocol as described previously [11].

### Oligomerization analysis of wild-type cHPL and cHPL mutants

The oligomerization state of 10–15 μg of a cHPL mutant or about 50 μg of wt cHPL was analyzed by size exclusion chromatography using a Superdex 200 Increase 10/300 GL (GE Healthcare) column with a 50 mM Tris (pH 8.0) and 150 mM NaCl buffer at a flow rate of 0.7 mL/min on an ÄKTA FPLC system (GE Healthcare) at 406 and 280 nm. The oligomerization states of the *C*. *imbricata* cAOS-LOX and the cAOS domain were determined in parallel. The retention times of the analyzed proteins were compared with authentic standards: thyroglobulin (669 kDa), ferritin (440 kDa), aldolase (158 kDa), BSA (67 kDa), ovalbumin (43 kDa), chymotrypsinogen (25 kDa), and ribonuclease A (13.7 kDa).

## Results

### The strategy for site-directed mutagenesis

**Fig 2:**
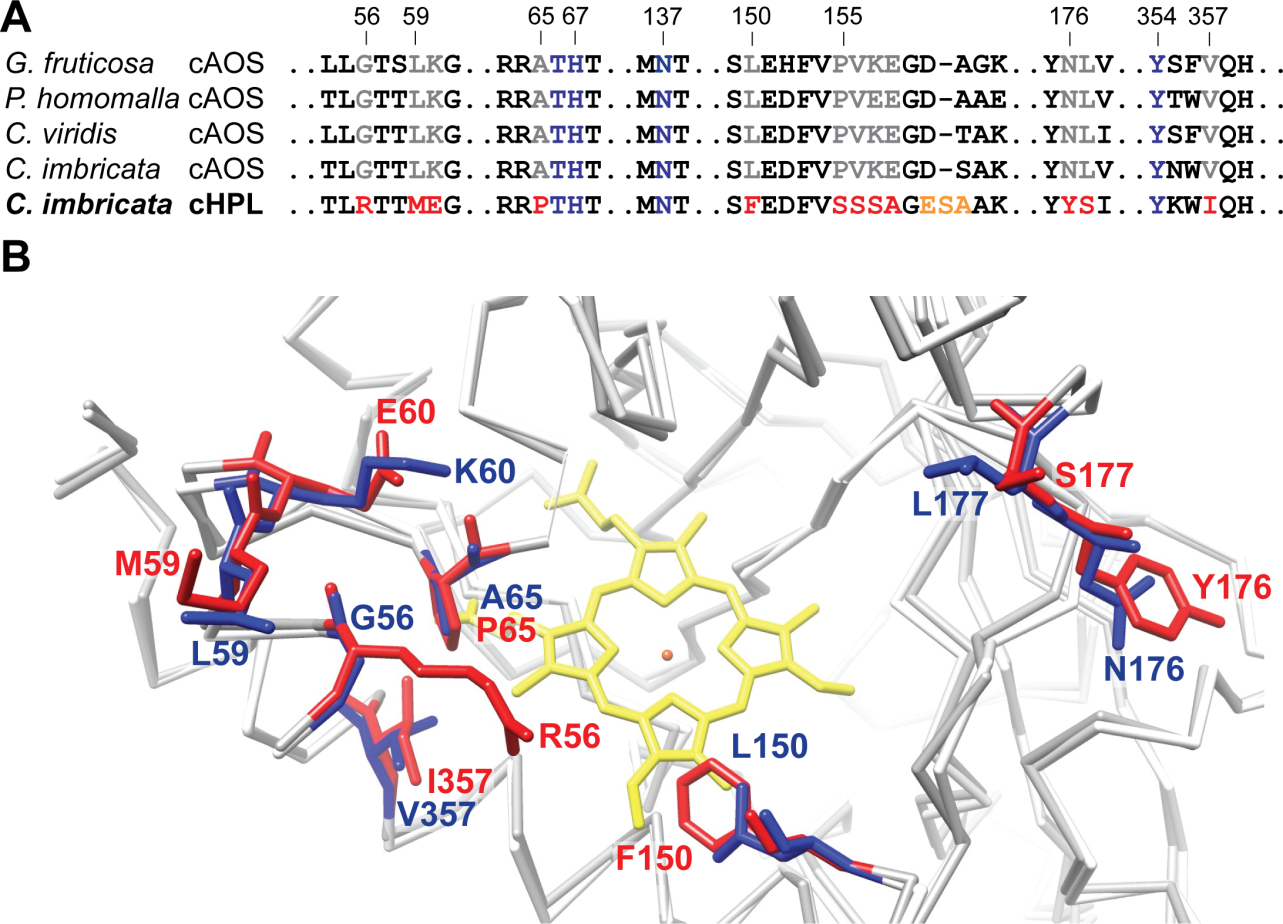
The differences between the residues of coral cAOSs and *C*. *imbricata* cHPL. **A**–alignment of amino acid sequences of coral cAOSs and *C*. *imbricata* cHPL presenting the conserved (blue) and distinct (grey vs red, respectively) residues in the substrate channel. The following amino acid sequences were compared: *Gersemia fruticosa* cAOS (NCBI ID: EU082210.1); *P*. *homomalla* cAOS (NCBI ID: AF003692.1); *Clavularia viridis* cAOS (NCBI ID: AB188528.1); *C*. *imbricata* cAOS (NCBI ID: KF000373.1); *C*. *imbricata* cHPL (NCBI ID: KF000374.1). **B–**the crystal structure of *P*. *homomalla* cAOS (blue) superpositioned with the model of *C*. *imbricata* cHPL (red) highlighting the main differences in the substrate pocket. The difference in the SSSAGE155-160PVKEGD fragments is not shown due to the illustrative purposes. The conserved amino acids between cAOS and cHPL are presented as a white and grey backbone, respectively. The heme is presented in yellow and the heme iron in orange.

**Fig 3:**
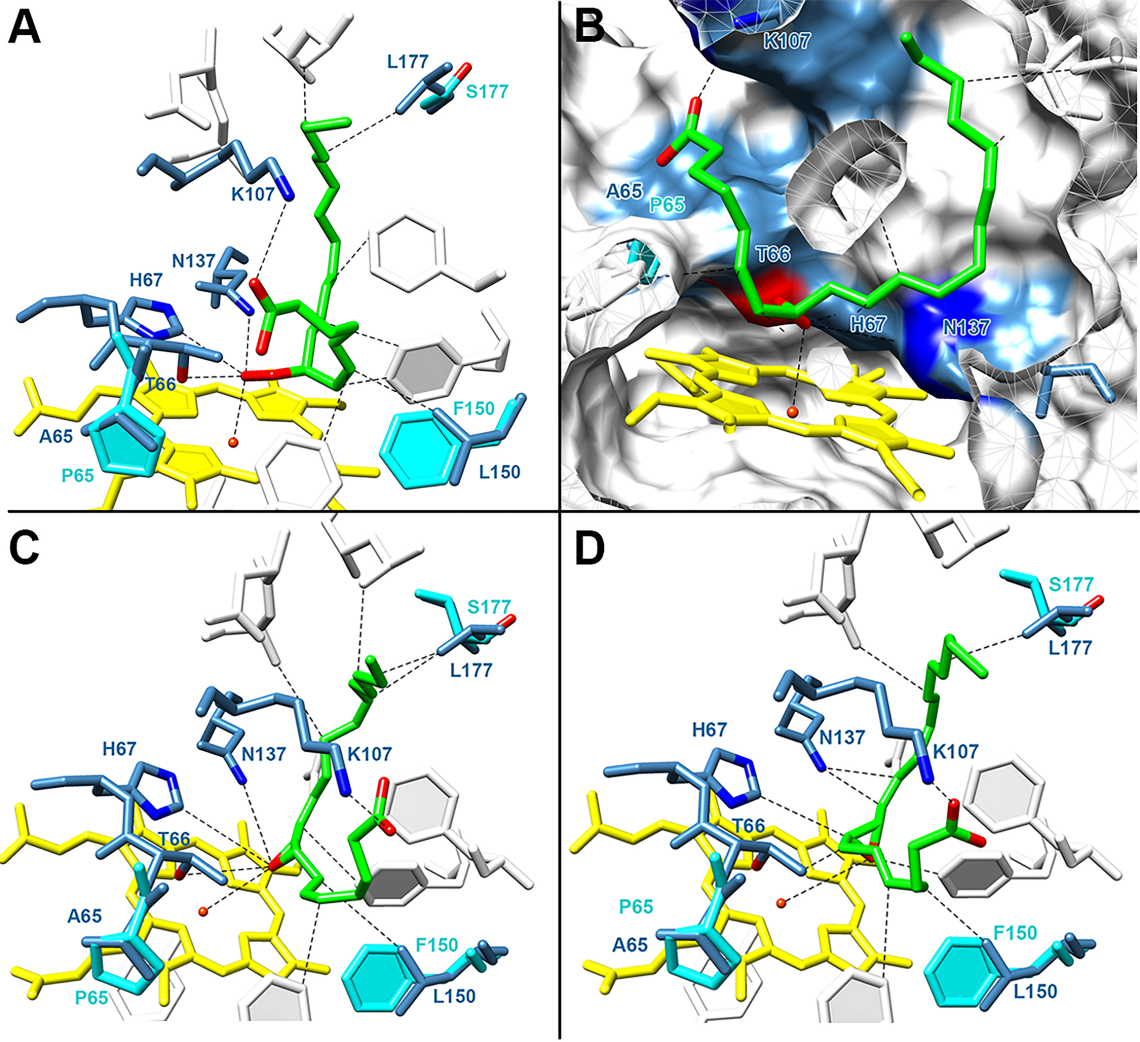
The docking analysis of *P*. *homomalla* cAOS with 8*R*-HpETE and AO. **A**– 8*R*-HpETE located in the substrate pocket; **B**–the rotated view of 8*R*-HpETE in the substrate pocket with a hydrophobic surface; **C**–AO in the substrate pocket; **D–**an alternative positioning of AO in the substrate pocket. The colors used in the figure are presented as follows: heme–yellow; heme iron–orange; ligands–green; the interacting residues of *P*. *homomalla* cAOS–blue; the interacting residues of *C*. *imbricata* cHPL–cyan; residues supporting the coordination of ligand or heme–gray; oxygen atoms–red; nitrogen atoms–blue. For clarity, the distances between the ligand and selected residues are given in the text.

To determine potential targets for mutational analysis, the residues of *C*. *imbricata* cHPL and coral cAOSs [9–11] were compared at sequence (Fig 2A) and structure levels (Fig 3A). Seven main differences in the residues located around the heme region, the substrate channel and the substrate entry site were observed as follows: P65A and I357V in the heme region; F150L in the connecting area between the heme and the substrate entry site (Fig 3A); YS176-177NL at the backside of the substrate channel coordinating the tail of the 8*R*-HpETE substrate (Fig 3A and 3B); R56G, ME59-60LK, and SSSAGE155-160PVKEGD in the active site entrance of the substrate channel. The amino acid pairs, i.e. R56/G56, P65/A65, F150/L150, and I357/V357, are highlighted in the superpositioned cHPL model and the crystal structure of *P*. *homomalla* cAOS, respectively (Fig 2B). All the presented substitutions in cHPL (Fig 2A) were performed and investigated. In addition, an opposite mutant, cAOS L150F, was prepared in parallel and will be discussed below.

### The coordination of ligands by cAOS and cHPL

Previous docking studies with *P*. *homomalla* cAOS have been focused only on the distal heme residues, H67 and T66, in regard of the interaction with a hydroperoxide [24]. As the main aim of this study was to determine the differences between the cHPL and cAOS substrate channels, we evaluated all the residues involved in the coordination of the 8*R*-HpETE substrate or AO.

The analysis of the X-ray structure of *P*. *homomalla* cAOS revealed that the negatively charged carboxy group of the 8*R*-HpETE substrate could be coordinated by the positively charged K107 and/or K60, located at the active site entrance of the substrate channel, and the hydroperoxy group of 8*R*-HpETE interacted with the catalytically important T66, H67, and N137 near the heme region [18]. These interactions were also observed in our docking analysis of *P*. *homomalla* cAOS with both ligands, 8*R*-HpETE and AO. As the length of H-bond should be less than 6 Å for stronger interaction [24], all the interactions determined in this study fulfilled this requirement.

Although the 8*R*-HpETE substrate was successfully placed inside the substrate channel of *P*. *homomalla* cAOS and *C*. *imbricata* cHPL models by the docking tool, the latter model did not give expected interactions between the distal heme residues, T66, H67, and the hydroperoxy group of 8*R*-HpETE. Docking analyses with individual cHPL mutants and also with the cHPL domain containing all the substituted residues did not give anticipated interactions either. Therefore, the further analysis was conducted only based on the docking results of *P*. *homomalla* cAOS.

The distance between the carboxy group of 8*R*-HpETE and K107 of cAOS was determined as 3.2 Å, while with K60 it was 5.0 Å. This may be indicative to the carboxy head coordination only by K107. The distances of T66, H67 and N137 from the hydroperoxy group of 8*R*-HpETE were determined as 3.0, 3.3 and 5.0 Å, respectively (Fig 3A and 3B). The weaker interaction between the N137 of cAOS and 8*R*-HpETE is in correlation with previous mutational studies of *P*. *homomalla* cAOS which indicated that the N137 was not important in the catalysis *per se*, but may have a supporting role in the substrate binding [25]. The docking with the second ligand, AO, showed that it can be located inside the substrate channel in two different conformations. In the first model, the epoxy group of AO was located closer to T66 and H67 (Fig 3C) with the respective distances of 4.7 and 4.5 Å. In the second model, the epoxy group was rotated 90 degrees away from T66 and H67 (Fig 3D) with distances of 5.5 and 5.2 Å, respectively. The distance between N137 and the epoxy group of AO, 4.6 Å, was the same for both models.

Among all the residues of our interest, the L150 and L177 of *P*. *homomalla* cAOS were the most proximal ones interacting with the U-shaped ligand (Fig 3). The corresponding residues in cHPL were F150 and S177, respectively. L150 was located near the C5-C7 of 8*R*-HpETE with the respective distances of 4.9, 3.4, 3.1 Å and was positioned on the opposite side of the hydroperoxy group (Fig 3A). L177 interacted with the hydrophobic tail at the C15-C17 of 8*R*-HpETE with distances of 3.3, 3.4, 4.0 Å, respectively (Fig 3A). The proximity between L150 and L177 with 8*R*-HpETE indicate that these residues are involved in the substrate binding and coordination.

### Protein expression and analysis of cHPL mutants

**Fig 4:**
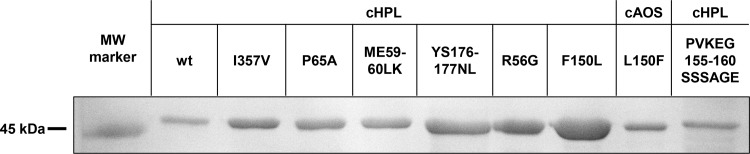
SDS-PAGE analysis of purified wt cHPL and corresponding mutants. Protein samples with an equal heme concentration (0.4 μM) were compared.

All the expressed mutants were determined to be catalytically active via enzyme assay using the 8*R*-HpETE substrate. Based on the obtained protein concentrations, the yields of cHPL R56G, ME59-60LK, P65A, YS176-177NL, and I357V mutants were determined to be 250–300 μg of soluble protein per 1 L of TB medium. The yields of wt cHPL, cHPL F150L, wt cAOS, and cAOS L150F were lower, 100, 170, 110, and 140 μg/L, respectively. However, the SSSAGE155-160PVKEGD alteration drastically reduced the yield of the corresponding mutant in the supernatant to 30 μg/L and therefore, size exclusion chromatography was not performed. The yields of wt cHPL and cHPL mutants were much lower compared to those of *P*. *homomalla* wt cAOS [26]. To estimate the presence of a heme, the protein samples at the same heme concentration were analyzed by SDS-PAGE (Fig 4). The heme content in wt cHPL and cHPL SSSAGE155-160PVKEGD samples was similar as there was no significant differences in the band sizes (Fig 4). The relative amount of heme-free protein in cHPL I357V, P65A, ME59-60LK, YS176-177NL, R56G, F150L, and cAOS L150F samples compared to the wt cHPL were determined as follows: 49%, 46%, 45%, 67%, 64%, 76% and 27%, respectively. In comparison with other samples, cHPL F150L sample contained the highest amount of heme-free protein (Fig 4) which implies notable changes in protein-heme interactions.

To investigate the influence of mutation on the Soret band of cHPL, the λ maxima of prepared mutants and wt enzymes were compared. In the UV spectra analysis, a 1 nm difference of the λ maximum of a heme between *C*. *imbricata* wt cAOS and cHPL was observed, 407 vs 406 nm, respectively. At the same time, the λ maxima of the N-terminal heme-containing domain and the corresponding fusion proteins were identical. The λ maxima of cHPL R56G and YS176-177NL remained unaltered. For other mutants, slight shifts of the Soret band at 405–408 nm were detected (data not shown) which can be explained by the small changes in the interactions between the heme, heme coordinating residues, proximal heme ligand, and proximal aromatic residues [27,28]. As there were no significant changes in the Soret bands, we can say that any of the mutations did not affect the conformation of the heme and the oxidation state of the heme iron [29–31].

**Fig 5:**
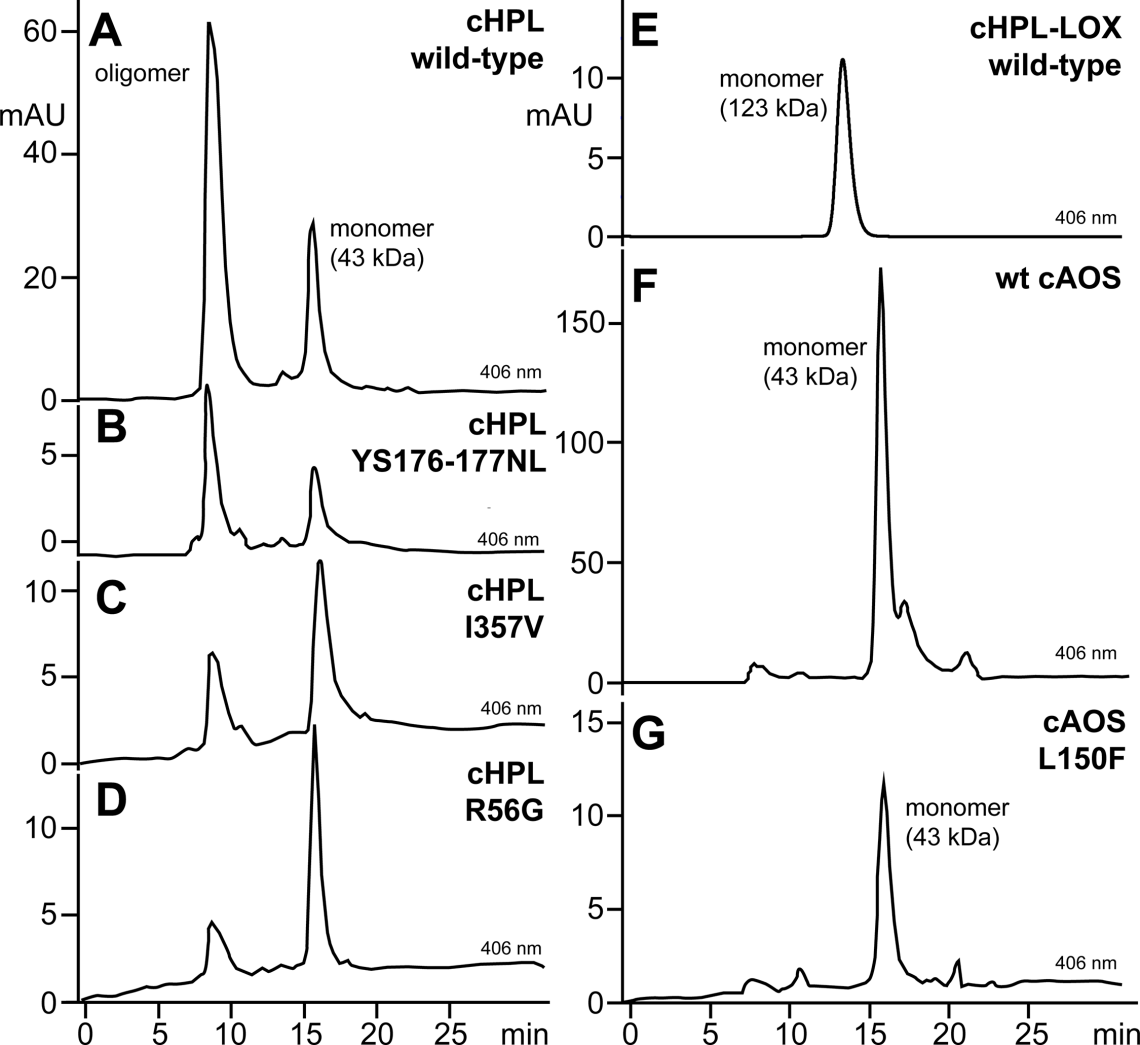
The oligomerization analysis of *C*. *imbricata* protein samples. **A**—wt cHPL; **B**—cHPL YS176-177NL. Similar oligomerization states were determined also for cHPL ME59-60LK, P65A, F150L. **C**—cHPL I357V; **D**—cHPL R56G; **E**—wt cHPL-LOX; **F**—wt cAOS; **G**—cAOS L150F. Oligomers and monomers of wt cHPL and mutants eluted at 8 and 16 min, respectively.

Size exclusion chromatography was performed to evaluate the influence of mutations on the oligomeric state of wt cHPL. The wt cHPL and cHPL ME59-60LK, P65A, F150L, and YS176-177NL mutants eluted from the size exclusion column as two peaks (Fig 5A and 5B). The higher early-eluting peak at 8 minutes represents oligomerized proteins with molecular weights at 440–670 kDa and the smaller peak at 16 minutes corresponds to the cHPL monomer at 45 kDa (Fig 5). The isolated monomer and oligomer maintained their oligomerization states after the reinjection on the size exclusion column. In parallel, the cHPL R56G and I357V mutants eluted mostly as monomers (Fig 5C and 5D). Both substitutions, R56G and I357V, are located in the α-helical part of cAOS which interacts with the LOX domain of the fusion protein [32]. Presumably, G56 and V357 influenced the properties of the corresponding α-helices and therefore, the oligomerization of the cHPL domain was reduced. In comparison, the *C*. *imbricata* cHPL-LOX (Fig 5E), the cAOS domain (Fig 5F), cAOS L150F (Fig 5G) and *P*. *homomalla* cAOS-LOX (S2 Fig) are monomers. However, the cause of the oligomerization of wt cHPL remains unclear.

### The activity of cHPL mutants

The determined k_cat_, K_m_ and k_cat_/K_m_ values of cHPL mutants with 8*R*-HpETE substrate are presented in Table 1. The corresponding kinetical parameters of wt cHPL were previously determined and resulted to be: 133.5 s^-1^, 3.8 μM, and 35.1 s^-1^ μM^-1^ [12]. The k_cat_ of cHPL F150L, P65A, and I357V mutants were 2-3-fold lower (Table 1) than that of wt cHPL. The major decrease in the k_cat_ value, 7.5 s^-1^, was detected with the ME59-60LK substitution. In contrast, R56G and YS176-177NL substitutions increased the k_cat_ approximately 3- and 2-folds, respectively. In the case of L150F cAOS, the k_cat_ was 7-fold lower, than those of wt cAOS and *P*. *homomalla* cAOS (Table 1). Due to the substrate inhibition of cHPL PVKEGD155-160SSSAGE with 8*R*-HpETE at higher substrate concentrations, the kinetic measurements were performed only with 5–30 μM 8*R*-HpETE substrate. The determined k_cat_ value of cHPL PVKEGD155-160SSSAGE, 52 s^-1^, was similar to those of cHPL F150L and P65A (Table 1).

**Table 1:** Kinetic parameters of wt cHPL, wt cAOS and selected mutants with 8*R*-HpETE and H_2_O_2_.

Enzyme		8*R*-HpETE			H_2_O_2_
		k_cat_ (s^-1^)	K_m_ (μM)	k_cat_/K_m_ (s^-1^, μM^-1^)	k_cat_ (s^-1^)
*C*. *imbricata cHPL*					
	**wild-type**	133.5 ± 5.0	3.8 ± 0.5	35.1	3
Heme region	**F150L**	49.6 ± 1.5	1.8 ± 0.4	27.6	14
	**P65A**	40.3 ± 2.5	15.3 ± 2.5	2.6	22
	**I357V**	63.9 ± 3.2	5.0 ± 1.0	12.8	8
Substrate channel	**R56G**	304.5 ± 22.8	19.0 ± 4.0	16.0	3
	**YS176-177NL**	218.1 ± 13.8	3.9 ± 0.9	56.0	31
	**ME59-60LK**	7.5 ± 0.6	7.3 ± 1.9	1.0	16
	**SSSAGE155-160PVKEG fragment**	52.1 ± 3.4	2.0 ± 0.8	26.1	35
*C*. *imbricata cAOS*					
	**wild-type**	1835.0 ± 196.3	46.6 ± 9.0	39.4	303
Heme region	**L150F**	203.3 ± 8.1	6.7 ± 1.0	30.3	127
*P*. homomalla cAOS*					
	**wild-type**	1409.2 ± 84.8	45.3 ± 7.5	31.1	insignificant^#^

The k_cat_ and K_m_ values determined with 8*R*-HpETE are presented with the corresponding standard error (n = 3). The k_cat_ values with H_2_O_2_ were determined as described in Materials and methods.

*—values obtained from the article by Boutaud *et al*. [26].

#—value obtained from the article by Tosha *et al*. [32].

The K_m_ values of cHPL F150L, I357V, YS176-177NL, ME59-60LK, PVKEGD155-160SSSAGE were comparable with those of wt cHPL. The remarkable 4- and 5-fold increase in K_m_ values was observed with cHPL P65A and R56G, respectively (Table 1). The K_m_ value of cAOS L150F was about 6-fold lower than those of wt cAOS and *P*. *homomalla* cAOS [26]. The elevated k_cat_ of cHPL R56G was probably achieved due to the increased accessibility of the substrate channel (S2 Table). However, the replacement of R56 with G56 lowered the affinity between 8*R*-HpETE substrate and the enzyme which indicates that R56 of wt cHPL is necessary for stronger binding of 8*R*-HpETE.

The catalytic efficiencies (k_cat_/K_m_) of cHPL P65A and ME59-60LK were determined to be more than 10-fold lower than the efficiency of the wt enzyme. The k_cat_/K_m_ of cHPL 1357V and R56G were about 2-fold lower whereas the catalytic efficiencies of F150L and PVKEGD155-160SSSAGE were comparable with wt cHPL’s. The reaction efficiency of cHPL YS176-177NL was about 2-fold higher than that of wt cHPL. The unexpected increase in the efficiency is commented in Discussion. The determined catalytic efficiency of cAOS L150F was in correlation with those of wt cAOS and *P*. *homomalla* cAOS (Table 1). All the mutations influenced the reaction rate and/or the affinity of cHPL with the 8*R*-HpETE substrate referring that all the substituted residues are necessary for the efficient catalysis.

To study the catalase activity of *C*. *imbricata* wt cHPL, wt cAOS and selected mutants, incubations with the H_2_O_2_ substrate were conducted. Wt cHPL and cHPL R56G had a very low reaction rate with H_2_O_2_, resulting in the initial production of 10 μM O_2_ per minute with the corresponding k_cat_ value of 2.5 s^-1^ which is in correlation with the measurements of *P*. *homomalla* cAOS [33]. With cHPL ME59-60LK, P65A, F150L, YS176-177NL, I357V, PVKEGD155-160SSSAGE, the oxygen levels increased up to 100 μM O_2_/min (k_cat_ = 8–35 s^-1^). In contrast, a considerably higher catalase activity was detected in the reactions using wt cAOS and cAOS L150F. The oxygen evolution by wt cAOS and cAOS L150F was determined as 1090 μM (k_cat_ = 303 s^-1^) and 450 μM O_2_/min (k_cat_ = 127 s^-1^), respectively. These values are about two orders of magnitude higher compared to wt cHPL’s. A similar catalase activity of *C*. *imbricata* cAOS-LOX to the individual cAOS domain was detected in parallel. In comparison with *P*. *homomalla* cAOS T66V [33], the oxygen evolution by *C*. *imbricata* cAOS was about 30-fold higher. *C*. *imbricata* and *P*. *homomalla* cAOSs share 82.8% of their amino acid sequences and there are no remarkable differences in the active site residues (Fig 1, S1A and S1B Fig), therefore, the higher ability of *C*. *imbricata* cAOS to break down H_2_O_2_ requires further investigation.

### Product synthesis by cHPL mutants

**Fig 6:**
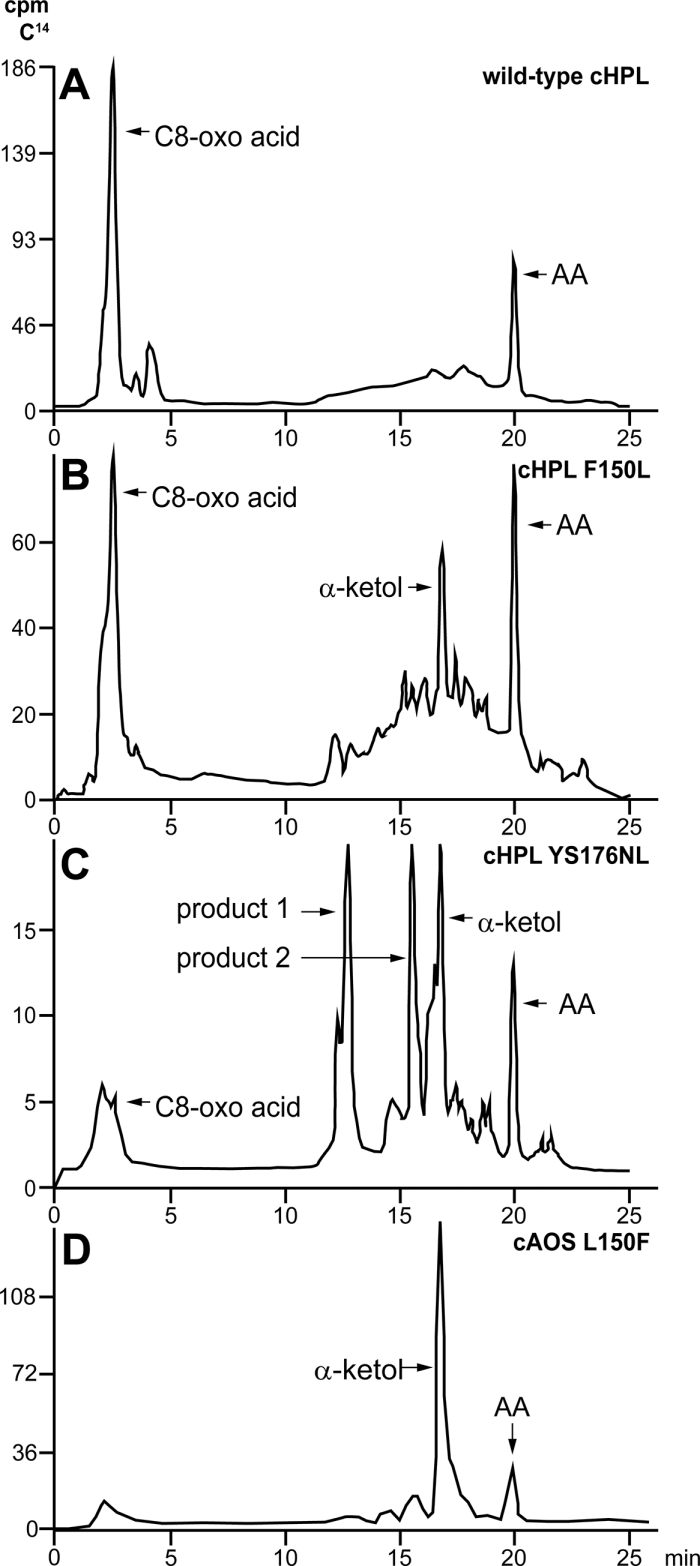
The products derived from the radiolabeled 8*R*-HpETE substrate by wt cHPL, selected cHPL mutants, and cAOS L150F. **A**– C8-oxo acid formed by wt cHPL representing also the products formed by cHPL R56G, P65A, ME59-60LK, and SSSAGE155-160PVKEG; **B**– C8-oxo acid and α-ketol formed by cHPL F150L; **C**– C8-oxo acid, α-ketol and product 1 and product 2 formed by cHPL YS176-177NL; **D**–α-ketol formed by cAOS L150F. The product pattern is identical to wt cAOS’s (data not shown).

Wt cHPL catalyzes the formation of C8-oxo acid and C12 aldehyde (Fig 1)[12]. On the radiochromatogram, C8-oxo acid, which retains the [1-^14^C] label, was the early-eluting peak at 2.5 minutes (Fig 6A). The detected product patterns of cHPL R56G, P65A, ME59-60LK, and SSSAGE155-160PVKEGD were identical to wt cHPL’s. In contrast to wt cHPL, cHPL F150L catalyzed the formation of C8-oxo acid and also α-ketol eluting at 2.5 and 17 min with conversion rates of 60% and 16% of total cpm value, respectively (Fig 6B). The cHPL YS176-177NL gave rise to the C8-oxo acid, α-ketol and also to two additional products, product 1 and 2. The conversion rates of corresponding products were determined as 18%, 9%, 46%, 15% (Fig 6C), respectively. The conversion rate to C8-oxo acid was determined as 18% whereas the corresponding value for α-ketol, product 1 and product 2 was about 23% (Fig 6C). Products 1 and 2 having the UV chromophore at 205 nm (data not shown) eluted at 12.5 and 16 min, respectively. The respective [M^-^] values of products 1 and 2 were determined to be 353 and 335 which correspond those of trihydroxy and epoxy alcohol derivatives, respectively. The product formation by cAOS was not altered by the opposite substitution, cAOS L150F, producing only the wt products, α-ketol and cyclopentenone [11], with elution times at 17 and 17.5 min, respectively (Fig 6D). The altered product profile and the docking analysis (see Results above) indicate that F150 and YS176-177 are involved in the correct substrate positioning.

## Discussion

**Fig 7:**
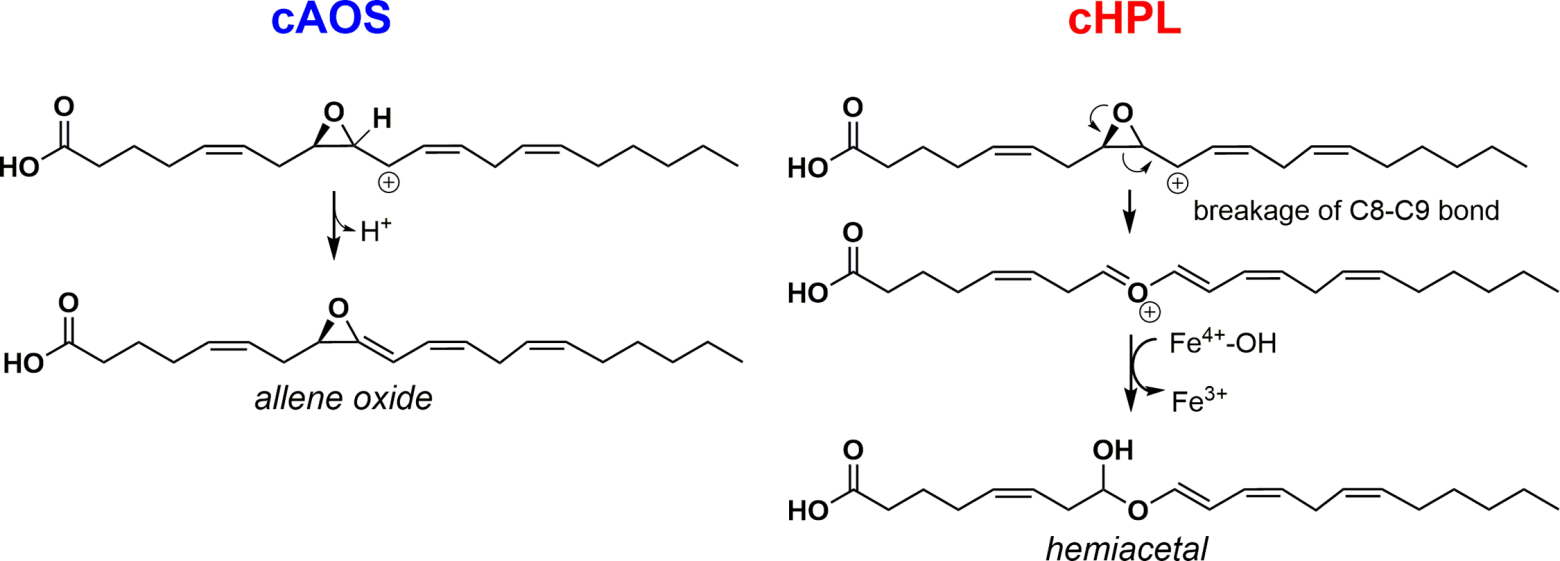
The proposed difference in reaction mechanisms between coral cAOS and cHPL. The hydrogen abstraction at C9 of the epoxy allylic carbocation is initiated by His67 of cAOS (blue). In cHPL-catalyzed reaction (red), no hydrogen abstraction occurs and instead, an unstable hemiacetal forms via the breakage of C8-C9 bond of the epoxide and the subsequent rebound of hydroxide.

Coral cAOS and cHPL catalyze the same initial steps until the formation of the 8*R*-HpETE-derived epoxy allylic radical (Fig 1). The further transformations of intermediates by cAOS and cHPL are controlled differently. In cAOS, H67 is involved in the initiation of the homolytic cleavage of hydroperoxide (Fig 1) and also in the abstraction of hydrogen from the C9 of the epoxy allylic carbocation resulting in the formation of AO [18](Fig 7). We suggest that in the cHPL-catalyzed mechanism, H67 might not be able to abstract hydrogen at C9 (Fig 7). Instead, the epoxy ring opening occurs via the breakage of C8-C9 bond resulting in the formation of an oxonium ion [34,35]. Next, the resonance structure of the oxonium ion, allylic ether carbocation, binds the hydroxide from the ferryl-hydroxo complex forming the highly unstable hemiacetal. In both reactions, the carbocation of cAOS- or cHPL-derived epoxy allylic intermediate [14,36] is stabilized at C10. In the cHPL-catalyzed reaction, the inability of H67 to abstract the hydrogen at C9 can be explained with the greater distance and/or unfavored positioning between the intermediate and H67. Moreover, there is no consensus describing AOS and HPL reactions in regard of ionic or radical intermediates. Both intermediates are possible, however, the most accepted way of presenting the AOS reaction is using a carbocation intermediate [14]. Therefore, for clarity in explaining the formation of multiple products from a common intermediate by cHPL F150L or YS176-177NL, only ionic intermediates were presented in Fig 7.

As suggested by Oldham *et al*. 2005, either the positively charged K107 or K60 could interact with the carboxy group of the 8*R*-HpETE substrate. K107 is present in the active site entrance of both enzymes, cAOS and cHPL. However, the 60th residue in cHPL is the negatively charged Glu (E60) which should have an opposite effect on the coordination of the negatively charged carboxy group of 8*R*-HpETE. In our study, the E60 of cHPL was replaced with the positively charged Lys (K60) of cAOS in regard to the double mutation, ME59-60LK, and instead of the expected improvement in activity and/or affinity, the reaction efficiency dropped remarkably. It indicates that although E60 in cHPL is necessary for the good activity of cHPL, the 60th residue might not be located near the carboxy head of 8*R*-HpETE. Instead, it could influence the reaction efficiency via conformational changes in the active site. As K107 of cAOS was located closer to the carboxy head of 8*R*-HpETE according to docking analysis, we postulate that K107 in cHPL and cAOS might be the main residue coordinating the carboxy group. However, the influence on the coordination of 8*R*-HpETE by K60 or K107 in cAOS and cHPL needs further investigation to confirm the obtained results. The positively charged R56 may also have an effect on the coordination of the carboxy head of 8*R*-HpETE. However, the replacement of R56 with G56 resulted in the unexpected increase in k_cat_ (Table 1) showing that R56 is not essential for the coordination of the carboxy group of 8*R*-HpETE and is also irrelevant in the matter of the cHPL-catalyzed reaction but seems to be necessary for stronger binding of the substrate.

Our data supports the hypothesis that the main differences between cHPL and cAOS in the interaction with a ligand were determined in the residues of F150/L150 and YS176-177/NL176-177 (cHPL/cAOS, respectively). Among other mutants, only the F150L and YS176-177NL substitutions resulted in the change of product pattern and influenced the catalysis process. A precedent can be found in the structural-functional study on the plant AOS where a single replacement of F to L resulted in the exchanged specificity from AOS to HPL [37]. As mentioned in the Introduction, cAOS and cHPL do not share any common structural elements with corresponding plant enzymes and therefore in coral isozymes, F150 and L150 may serve a different role in the reaction with the 8*R*-HpETE substrate. Based on ligand binding analysis, L150 of *P*. *homomalla* cAOS interacts with the C5-C6 double bond of 8*R*-HpETE (Fig 3A) and thus the corresponding residue in cHPL, bulkier F150, might direct the epoxy allylic carbocation into a position where the hydrogen abstraction at C9 is not possible (Fig 7). The opposite mutation in cAOS, L150F, did not cause any change in the product profile, indicative that the corresponding residue in cAOS, L150, is not directly involved in the catalysis. A similar phenomenon can be observed in the structural-functional analysis of the plant enzymes where the mutations between the Cyp74 family members, AOS, HPL or DES, were not interchangeable [38,39]. Moreover, the inability of cAOS L150F to synthesize aldehydes might be explained by the proposed theory about the evolution of plant AOS from HPL [38]. Based on our results, the distinct effect of a mutation on product formation implies that L150 of cAOS and F150 of cHPL interact with the substrate differently due to slight variances in the active site and/or the positioning of the substrate.

According to our docking analysis, the L177 of *P*. *homomalla* cAOS interacted with the aliphatic tail of 8*R*-HpETE. We suggest that as a result of the YS176-177NL mutation, the repositioning of the epoxy allylic radical intermediate took place which resulted in the increased activity of cHPL and in the production of multiple products. The formation of unexpected products (Fig 6C) can be explained by the uncontrolled catalysis. Presumably, the initial step in the reaction remained unaltered but the further intramolecular rearrangements of the epoxy allylic radical were taking different directions as it has been described with the formation of AO, hemiacetal, divinyl ether or epoxy alcohol by the closely related enzymes of the Cyp74 family [14]. The increase in the activity of cHPL YS176-177NL cannot be explained by the change of the volume of substrate channel as it is nearly identical with wt cHPL’s (S2 Table). Probably, the nonpolar properties of NL instead of polar YS provided stronger interactions with the hydrophobic tail of 8*R*-HpETE and promoted a faster reaction rate by directing the substrate in a more favorable position. As with both mutants, F150L and YS176-177NL, there was no total shift in the activity from cHPL to cAOS, we suggest that alternative conformations of the intermediate in the active site were possible. Therefore, the capability of corresponding cHPL mutants to abstract hydrogens by H67 was dependent on the conformation of the intermediate.

In the current study, we elucidated how the site-specific mutations in the substrate channel influenced the reaction efficiency, specificity and oligomerization of *C*. *imbricata* cHPL. Based on the altered product formation, F150 and YS176-177 were established as the reaction-specific residues of cHPL. As the determinants of the reaction mechanism and the substrate coordination of coral cHPL and cAOS are not fully understood, additional structural-functional studies are a matter of future research.

## Supporting information

S1 Table Forward and reverse primers of *C. imbricata* cHPL mutants, wt cAOS domain, cAOS L150F and 8*R*-LOX domain. (PDF)

S2 Table The surface volume and area analysis of *C. imbricata* wt cHPL, wt cAOS, *P. homomalla* cAOS and selected mutants. (PDF)

S1 Fig The sequence comparison of *P. homomalla* cAOS, *C. imbricata* cHPL domain. **A—**the amino acid identity and divergence between *P*. *homomalla* cAOS domain, *C*. *Imbricata* cHPL domain and mutants; **B**—the sequence alignment of *P*. *homomalla* cAOS and *C*. *imbricata* cAOS.
(PDF)

S2 Fig The oligomerization state of *P. homomalla* cAOS-LOX. Size exclusion chromatography was performed the same way as described in Materials and Methods.
(PDF)

S1 File The homology model of *C. imbricata* cHPL. The model was prepared based on the X-ray structures of *P*. *homomalla* cAOS (PDB IDs: 1u5u and 3dy5) using the CPHmodels-3.2 server.
(PDB)

S1 Text The topography of *C. imbricata* cHPL. (PDF)

## Abbreviations

AA: arachidonic acid
AO: allene oxide
cAOS: catalase-related allene oxide synthase
cHPL: catalase-related hydroperoxide lyase
HpETE: hydroperoxy-eicosatetraenoic acid
LOX: lipoxygenase
wt: wild-type
