# Supplementary material for: Structural and functional insights into the reaction specificity of catalase-related hydroperoxide lyase: A shift from lyase activity to allene oxide synthase by site-directed mutagenesis
Source: PLoS One. 2017 Sep 27;12(9):e0185291. doi: 10.1371/journal.pone.0185291 (PMC5617202; doi:10.1371/journal.pone.0185291)
Supplement: S1 Table — (PDF) [file pone.0185291.s001.pdf]

S1 Table

**Enzyme**	**Primers (5' – 3')**
cHPL-H4 R56G	*Up* (*Nco*I) *:* CCTCGGTACAACCATGGAAGGATTTC<br>*Down* (*Nco*I)*:* GAAATCCTTCCATGGTTGTACCGAGGG
cHPL-H4 ME59-60LK	*Up* (*Pfl23*II)*:* ACCCTCCGTACGACGTTGAAAGGAT<br>*Down* (*Pfl23*II)*:* GAAATCCTTTCAACGTCGTACGG
cHPL-H4 P65A	*Up* (*Kpn2*I)*:* AAGGATTCCGGAGAGCAACCC<br>*Down* (*Kpn2*I)*:* GTGGGTTGCTCTCCGGAATCC
cHPL-H4 F150L	*Up* (*Xho*I)*:* CTCGAGCTTGGAGGACTTTGTATC<br>*Down* (*Xho*I)*:* GATACAAAGTCCTCCAAGCTCGAG
cHPL-H4 YS176-177NL	*Up* (*Hind*III)*:* TATTACAATTTAATCGAAGCTTTA CGACGTGC<br>*Down* (*Hind*III)*:* CGTAAAGCTTCGATTAAATTGTAATAATAATAAGG
cHPL-H4 I357V	*Up* (*Kpn2*I)*:* GTGTACAAATGGGTGCAGCATCTTC<br>*Down* (*Kpn2*I)*:* GAAGATGCTGCACCCATTTGTACAC
cHPL-H4 PVKEGD155-160SSSAGE	Insertion of *XhoI* restriction site:<br>*Up:* TCTGGAACACCTCGAGCTTTGAGG<br>*Down:* GTCCTCAAAGCTCGAGGTGTTCC<br>Fragment replacement:<br>*Up (XhoI):* CACACCTCGAGCTTTGAAGACTTTGTAC<br>*Down (PsiI):* ATTCTTATAAACATATTCCTTTGCAGAATCGCC
wt cAOS-H4	*Up (NheI):* AATGCTAGCATGACTTGGAAAAATTTTGGATTTGATATC<br>*Down (NheI):*<br>ATAGCTAGCTTAGTGATGGTGATGGTTCTGTCCAGCTGGAATCAGAC
cAOS-H4 L150F	*Up (XhoI):* CACCTCGAGCTTTGAAGACTTTG<br>*Down (XhoI):* GTCTTCAAAGCTCGAGGTGTGC
His_6-8*R*-LOX domain of cHPL-LOX	*Up (NheI):*<br>ATTCATATGCATCACCATCACCATCAC<br>GCACTGTATAATATTGAAGTTG<br>*Down (NdeI):*<br>CAGCTAGCCTAGATTGCAGTTCCGTTAGG
