# Supplementary material for: Structural and functional insights into the reaction specificity of catalase-related hydroperoxide lyase: A shift from lyase activity to allene oxide synthase by site-directed mutagenesis
Source: PLoS One. 2017 Sep 27;12(9):e0185291. doi: 10.1371/journal.pone.0185291 (PMC5617202; doi:10.1371/journal.pone.0185291)
Supplement: S2 Table — (PDF) [file pone.0185291.s002.pdf]

S2 Table

Enzyme	Pocket volume (Å^3)[1]	Pocket area (Å^2)	Mouth area (Å^2)
C. imbricata			
**wt cHPL**	1147,4	1425	67
**R56G**	1103,4	1357	83
**ME59-60LK**	1116,4	1388	64
**P65A**	1290,4	1566	139
**F150L**	1182,4	1461	112
**YS176-177NL**	1143,4	1430	102
**I357V**	1267,4	1612	110
**PVKEG**	1216,4	1442	60
**wt cAOS**	1268,4	1487	80
**L150F**	1353,4	1553	98
P. homomalla			
**cAOS**[2]	1802,4	1743	98

[1] - the volume of the heme was excluded from the calculations of the pocket volume

[2] - the volume and the area of substrate pocket of *P. homomalla* cAOS was calculated based on the crystal structure its crystal structure (PDB ID: 1u5u)(Oldham *et al.* 2015).
