# Supplementary material for: Structural and functional insights into the reaction specificity of catalase-related hydroperoxide lyase: A shift from lyase activity to allene oxide synthase by site-directed mutagenesis
Source: PLoS One. 2017 Sep 27;12(9):e0185291. doi: 10.1371/journal.pone.0185291 (PMC5617202; doi:10.1371/journal.pone.0185291)
Supplement: S1 Fig — A—the amino acid identity and divergence between P. homomalla cAOS domain, C. Imbricata cHPL domain and mutants; B—the sequence alignment of P. homomalla cAOS and C. imbricata cAOS. (PDF) [file pone.0185291.s003.pdf]

S1 Fig

A – the amino acid identity and divergence between *P. homomalla* cAOS domain, *C. Imbricata* cHPL domain and mutants.

Percent Identity (upper triangle) / Divergence (lower triangle)

	1	2	3	4	5	6	7	8	9		
1		82.8	83.1	83.4	83.1	83.1	83.9	83.4	83.1	1	cAOS Plexaura homomalla.pro
2	19.5		99.7	99.5	99.7	99.7	98.7	99.5	99.7	2	cHPL Capnella imbricata.pro
3	19.2	0.3		99.2	99.5	99.5	98.4	99.2	99.5	3	cHPL R56G.pro
4	18.8	0.5	0.8		99.2	99.2	98.1	98.9	99.2	4	cHPL ME59-60LK.pro
5	19.2	0.3	0.5	0.8		99.5	98.4	99.2	99.5	5	cHPL P65A.pro
6	19.2	0.3	0.5	0.8	0.5		98.4	99.2	99.5	6	cHPL F150L.pro
7	18.2	1.3	1.6	1.9	1.6	1.6		98.1	98.4	7	cHPL SSSAGE155-160PVKEGD.pro
8	18.8	0.5	0.8	1.1	0.8	0.8	1.9		99.2	8	cHPL YS176-177NL.pro
9	19.2	0.3	0.5	0.8	0.5	0.5	1.6	0.8		9	cHPL I357V.pro
	1	2	3	4	5	6	7	8	9		

B – the sequence alignment of *P. homomalla* cAOS and *C. imbricata* cAOS.

```
Majority                      MTWKNFGFDIFGEKFGQDELEKRIKDEHTPPPDSPVFGGLTLKLKIEKFKTLFTLGTTLKGFRRATHTVGTGGIGEVTVV
                                       10        20        30        40        50        60        70        80
cAOS Plexaura homomalla.pro   MTWKNFGFEIFGEKYGQEELEKRIKDEHTPPPDSPVFGGLKLKLKKEKFKTLFTLGTTLKGFRRATHTVGTGGIGEITIV  80
cAOS Capnella imbricata.pro   MTWKNFGFDIFAERFGKDKLEKRIKDERTPPPDSPVFGGLTLRMKIEKFKTLFTLGTTLKGFRRATHTVGTGGIGEVTVV  80

Majority                      DDPKFPEHEFFTAGRTLPARLRHANLKYPDDAGADARSFSIKFADSDSDGPLDIVMNTGEANIFWNSSSLEDFVPVEEGD
                                       90       100       110       120       130       140       150       160
cAOS Plexaura homomalla.pro   NDPKFPEHEFFTAGRTFPARLRHANLKYPDDAGADARSFSIKFADSDSDGPLDIVMNTGEANIFWNSPSLEDFVPVEEGD  160
cAOS Capnella imbricata.pro   DDPKFPEHEFFKAGRKLPVRLRHANLKYPDDAGADARSFSIKFSDNDSESPLDIVMNTGEANIFWHTSSLEDFVPVKEGD  160

Majority                      AAEEYVYKNPYYYYNLVEALRRAPDTFAHLYYYSQVTMHFKAKDGKVRYCRYRALPGDVDIKEEDESGRLTEEEQRNIWI
                                      170       180       190       200       210       220       230       240
cAOS Plexaura homomalla.pro   AAEEYVYKNPYYYYNLVEALRRAPDTFAHLYYYSQVTMPFKAKDGKVRYCRYRALPGDVDIKEEDESGRLTEEEQRKIWI  240
cAOS Capnella imbricata.pro   SAKEYVYKNPYYYYNLVEALRRAPNTFAHLYYYSQVTMHFKAKDGKERYCRYRAIPGDVDIKEEEESGRLTEEEQRNIWT  240

Majority                      FSRHETEKRPDDYLRKEYVERLQKGPVNYRLQIQIHDASPDDTATIFHAGILWDKETHPWFDLAKVSITTPLSPDVLEKT
                                      250       260       270       280       290       300       310       320
cAOS Plexaura homomalla.pro   FSRHENEKRPDDYLRKEYVERLQKGPVNYRLQIQIHEASPDDTATIFHAGILWDKETHPWFDLAKVSIKTPLSPDVLEKT  320
cAOS Capnella imbricata.pro   FSRYETEKRPDDYLRKEYVERLKKGPVNYRLQIQIHDVSPNDTATIFHAGILWDKETHPWFDLAKVSITTPMSPDVLERT  320

Majority                      AFNIANQPASLGLLDAKSPEDYNSIGELRVAVYTWVQHLRKLKLGSLVPAGQN
                                      330       340       350       360       370
cAOS Plexaura homomalla.pro   AFNIANQPASLGLLEAKSPEDYNSIGELRVAVYTWVQHLRKLKIGSLVPAGQN                             373
cAOS Capnella imbricata.pro   NFNIVNQPKSLGLLDAKSPEDYNSIGQIRVSVYNWVQHLRKLKLGSLIPAGQN                             373
```
