# Supplementary figures and images for: Structural and functional insights into the reaction specificity of catalase-related hydroperoxide lyase: A shift from lyase activity to allene oxide synthase by site-directed mutagenesis
Source: PLoS One. 2017 Sep 27;12(9):e0185291. doi: 10.1371/journal.pone.0185291 (PMC5617202; doi:10.1371/journal.pone.0185291)

**S2 Fig**

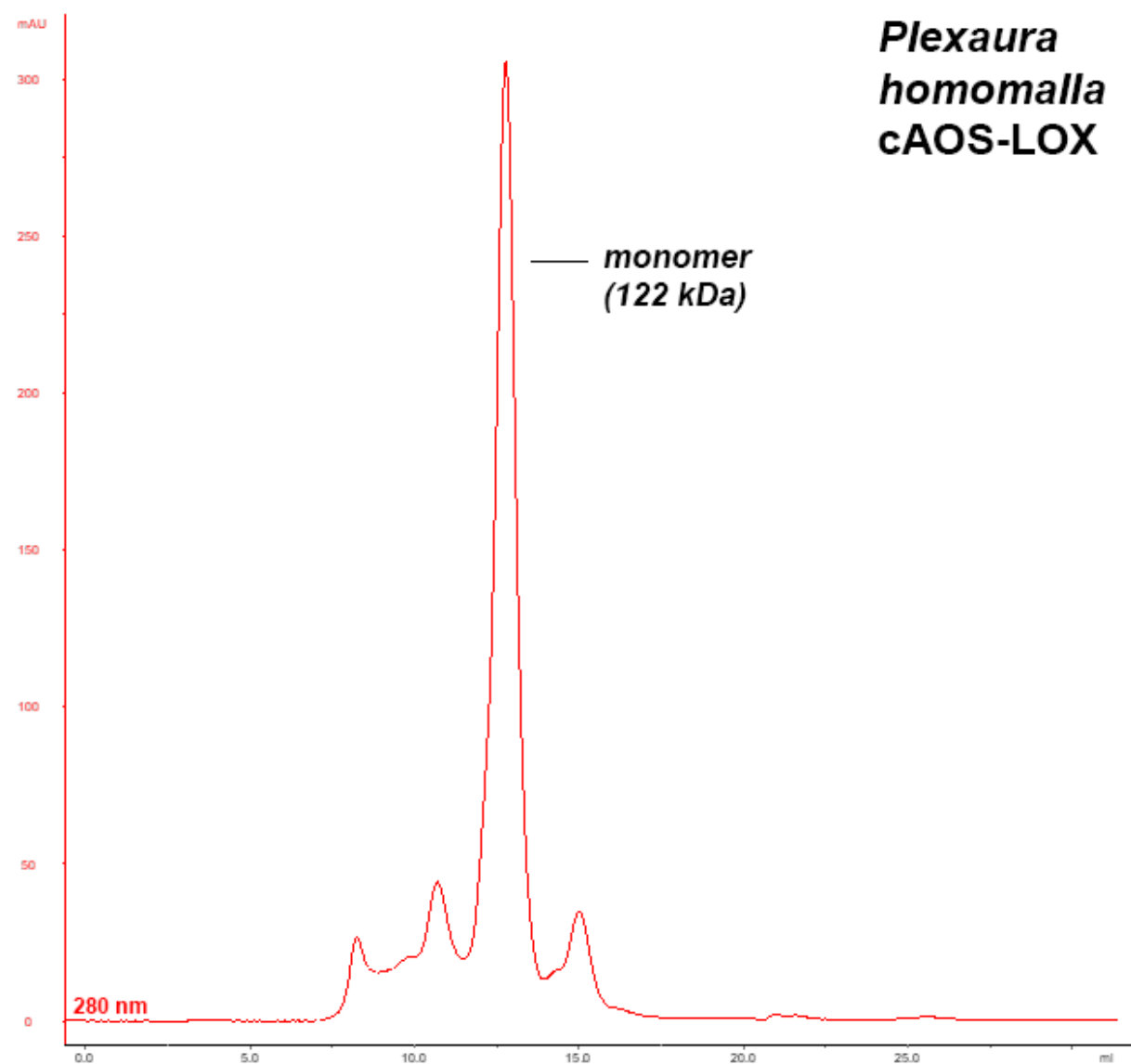

Supplement: S2 Fig — Size exclusion chromatography was performed the same way as described in Materials and Methods. (PDF) [file pone.0185291.s004.pdf]
