# Supplementary material for: Structural and functional insights into the reaction specificity of catalase-related hydroperoxide lyase: A shift from lyase activity to allene oxide synthase by site-directed mutagenesis
Source: PLoS One. 2017 Sep 27;12(9):e0185291. doi: 10.1371/journal.pone.0185291 (PMC5617202; doi:10.1371/journal.pone.0185291)
Supplement: S1 Text — (PDF) [file pone.0185291.s006.pdf]

S1 Text

The volume of the heme was determined to be 510.6 $Å^3$ and was excluded from the total volume of the substrate channel in further calculations. The surface area and volume of wt cHPL were determined as 1425 $Å^2$ and 1147 $Å^3$ with the mouth area of 67 $Å^2$ (S2 Table). The corresponding values for *P. homomalla* cAOS substrate pocket were 1743 $Å^2$ and 1802 $Å^3$ including 98 $Å^2$ of themouth area. The *C. imbricata* cAOS has a similar substrate binding pocket and mouth area but about 100 $Å^3$ larger pocket volume compared to the *C. imbricata* cHPL's. The volume of substrate channel of cAOS was greater due to the wider mouth area and a side-pocket below the heme (data not shown). The residues that determined the volume and the area of binding pockets by CASTp server also contained the substituted amino acids used in this study (data not shown). The calculated pocket volumes and areas of cHPL mutants and cAOS L150F are presented in S2 Table. The pocket area and volume of cHPL R56G, ME59-60LK, F150L, YS176-177NL, and SSSAGE155-160PVKEGD were similar to those of wt cHPL. However, the volume and area of substrate channels of cHPL P65A and I357V were about 200 units higher. The mouth areas of cHPL R56G, ME59-60LK, SSSAGE155-160PVKEGD and wt cHPL were similar, between 60-80 $Å^2$. The mouth area larger than 100 $Å^2$ was calculated for P65A, F150L, YS176-177NL and I357V. The substitution of L150F in cAOS resulted in the significant increase of pocket volume, pocket area and mouth area compared to wt cAOS's.
